# The effect of mannitol on intraoperative brain relaxation in patients undergoing supratentorial tumor surgery: study protocol for a randomized controlled trial

**DOI:** 10.1186/1745-6215-15-165

**Published:** 2014-05-10

**Authors:** Yuming Peng, Xiaoyuan Liu, Aidong Wang, Ruquan Han

**Affiliations:** 1 Department of Anesthesiology, Beijing Tiantan Hospital, Capital Medical University, No. 6, Tiantan Xili, Dongcheng District, Beijing, 100050, PR China

**Keywords:** Randomized controlled trial, Mannitol, Brain relaxation, Supratentorial tumor

## Abstract

**Background:**

The risk of brain swelling after dural opening is high in patients with midline shift undergoing supratentorial tumor surgery. Brain swelling may result in increased intracranial pressure, impeded tumor exposure, and adverse outcomes. Mannitol is recommended as a first-line dehydration treatment to reduce brain edema and enable brain relaxation during neurosurgery. Research has indicated that mannitol enhanced brain relaxation in patients undergoing supratentorial tumor surgery; however, these results need further confirmation, and the optimal mannitol dose has not yet been established. We propose to examine whether different doses of 20% mannitol improve brain relaxation in a dose-dependent manner when administered at the time of incision. We will examine patients with preexisting mass effects and midline shift undergoing elective supratentorial brain tumor surgery.

**Methods:**

This is a single-center, randomized controlled, parallel group trial that will be carried out at Beijing Tiantan Hospital, Capital Medical University. Randomization will be achieved using a computer-generated table. The study will include 220 patients undergoing supratentorial tumor surgery whose preoperative computed tomography/magnetic resonance imaging results indicate a brain midline shift. Patients in group A, group B, and group C will receive dehydration treatment at incision with 20% mannitol solutions of 0.7, 1.0, and 1.4 g/kg, respectively, at a rate of 600 mL/h. The patients in the control group will not receive mannitol. The primary outcome is an improvement in intraoperative brain relaxation and dura tension after dehydration with mannitol. Secondary outcomes are postoperative outcomes and the incidence of mannitol side effects.

**Discussion:**

The aim of this study is to determine the optimal dose of 20% mannitol for intraoperative infusion. We will examine brain relaxation and outcome in patients undergoing supratentorial tumor surgery. If our results are positive, the study will indicate the optimal dose of mannitol to improve brain relaxation and avoid side effects during brain tumor surgery.

**Trial registration:**

The study is registered with the registry website http://www.chictr.org with the registration number ChiCTRTRC13003984 (17 December 2013).

## Background

The average incidence of intracranial tumors in China is ten per hundred thousand healthy people. Most of these tumors are supratentorial tumors (79%), the most common of which are gliomas, meningiomas, and pituitary tumors. Supratentorial tumors produce significant mass effects in the brain, and certain types are accompanied by significant peritumoral edema that leads to increased intracranial pressure. Bedford et al. [1] showed that the preoperative degree of peritumoral edema was closely related to the postoperative increase in intracranial pressure. Rasmussen et al. [2] revealed that the risk of brain swelling after dura opening was very high in 692 cases of patients with glioma with midline shift undergoing craniotomy; this result indicated that in cases with preoperative increased intracranial pressure, dehydration treatment should be administered before cutting the dura. Dehydration aids in ensuring proper brain relaxation and facilitates tumor exposure.

Higher osmotic pressure in the blood vessels after the infusion of mannitol drives water molecules from the brain tissue to blood vessels and results in brain tissue dehydration. However, the role of mannitol in reducing brain edema depends on an intact blood–brain barrier (BBB). If the BBB is damaged, mannitol will extravasate outside the blood vessels and will transfer water molecules into brain tissue, which will aggravate cerebral edema and increase intracranial pressure [3]. There may be some degree of BBB disruption in certain patients, which would prevent the desirable effects of mannitol; however, the extent of this disruption is unclear and often affected by multiple-dose mannitol [4]. The use of mannitol for the type of surgery that patients in our study will undergo has been found overall to be beneficial; however, the appropriate dose of mannitol is controversial, particularly since large multiple doses can have negative effects. The point of our study is to determine a dose that leads to a beneficial effect without triggering negative effects. Some clinicians [5,6] advocate high doses (>1.0 g/kg) of mannitol to effectively reduce intracranial pressure, while others recommend lower doses (<1.0 g/kg) [7,8]. Treatment guidelines for using mannitol in patients with traumatic brain injury and stroke have been published and provide recommendations regarding the dose and timing of mannitol. However, there is still controversy concerning dehydration treatment with mannitol in patients with preoperatively increased intracranial pressure during brain tumor surgery.

The effect of mannitol on intraoperative brain relaxation has been increasingly studied in recent years. In 2007, Rozet et al. [9] compared the effects of mannitol and hypertonic saline on intraoperative brain relaxation in 40 patients undergoing elective craniotomy and found a similar effect in both treatment groups. However, the intracerebral pathology of the patients in this study varied widely, and only six of the ten patients with supratentorial brain tumors received mannitol. Additionally, the preoperative peritumoral edema and intracranial pressure were not recorded, and only a single dose of mannitol (1 g/kg) was administered. Wu et al. [10] compared the effects of 160 mL of 3% hypertonic saline and 150 mL of 20% mannitol on brain relaxation. Their study suggested that 3% hypertonic saline provided better relaxation; however, the lengths of hospital and intensive care unit stays did not significantly differ. The dose of mannitol administered in the study was not adjusted according to the preoperative intracranial pressure, the intracranial mass occupying effect, or the body weight of the patient. Furthermore, the dose of mannitol was lower than the commonly used clinical dose. Therefore, this study is of little use as a clinical guide. More recently, a prospective randomized controlled study [11] demonstrated that a single dose of 0.7 or 1.4 g/kg mannitol achieved similar brain relaxation in patients undergoing craniotomy and tumor resection. However, further statistical analysis that took into account the preoperative midline shift indicated that the high dose yielded a better outcome. The study indicated that the effect of mannitol on brain relaxation may be dose-dependent if the preoperative increase in intracranial pressure is taken into consideration; however, further study is required to verify this suggestion.

Mannitol and hypertonic saline are often used as dehydrating agents in neurosurgery and neurology. Hypertonic saline has been widely used in patients with stroke and traumatic brain injury to reduce brain edema and intracranial pressure; however, its use, especially during the operation, remains controversial in patients undergoing brain tumor surgery. Starke et al. [12] presented a comprehensive assessment of the effect of hypertonic saline during brain tumor resection and did not recommend it as a treatment for dehydration in brain tumor surgery unless the patient presents with hyponatremia and hypotension. Dehydration treatment with mannitol was recommended to reduce brain edema and provide brain relaxation during neurosurgery.

The above series of randomized control trials aimed to assess the effect of mannitol on brain edema and relaxation; however, there were limitations associated with their conclusions. First, the studies did not describe preoperative factors that may affect brain relaxation, such as the size and histological type of the tumor, peritumoral edema [2], the position of the head and body, body temperature, and arterial carbon dioxide partial pressure. Second, the studies did not indicate whether high-risk patients with increased brain pressure were included. Finally, these studies lacked data comparing the clinical side effects and patient outcomes following the infusion of mannitol.

Based on the current literature, we propose that different doses of mannitol will improve brain relaxation, as well as ease surgical exposure in a dose-dependent manner for patients with preoperative midline shift undergoing elective supratentorial brain tumor surgery. A double-blind randomized controlled trial of intraoperative intravenous mannitol in patients undergoing supratentorial tumor surgery will be conducted to examine this hypothesis.

## Methods/design

### Study objectives

#### Primary aim

The primary aim of our trial is to investigate whether the intraoperative administration of 0.7, 1.0, and 1.4 g/kg body weight intravenous mannitol at a rate of 600 mL/h results in an improvement of brain relaxation in patients with a supratentorial brain tumor. Brain relaxation will be scored by the surgeon, who is blinded to the treatment, upon opening the dura using a four-point scale: 1 = completely relaxed, 2 = satisfactorily relaxed, 3 = firm brain, 4 = bulging brain [13].

#### Secondary aim

The secondary aim is to determine whether different doses of intraoperative mannitol alter hemodynamic parameters, plasma electrolytes, blood glucose, and lactic acid such that the perioperative outcomes of patients are improved. Additionally, we want to determine whether different doses of intraoperative mannitol alter patients’ three-month postoperative outcomes, including cerebral edema, cerebral hemorrhage, recurrence, and death.

### Anesthesia depth measurement

We will measure the mean blood pressure, heart rate, bispectral index, end-tidal carbon dioxide, and inspiratory and expiratory gas concentrations and will maintain a similar anesthesia depth. The mean arterial pressure will be maintained between 10% below and 20% above the baseline value. Anesthetic depth will be determined by using the bispectral index (Covidien LLC, USA), which will be maintained between 40 and 60.

### Study design

This is a prospective, single-center, randomized, parallel group controlled trial that is being conducted at Beijing Tiantan Hospital, Capital Medical University, Beijing, China. Study recruitment commenced on 1 January 2014. Recruitment is expected to take 12 months. There are more than 1,000 cases of supratentorial tumor surgery per year at Beijing Tiantan Hospital, Capital Medical University. We plan to complete the study in one year.

### Randomization and blinding

Randomization will be conducted via a computer-produced randomized controlled table. Participants will be randomized within 24 hours prior to surgery. A research nurse will give the participants mannitol or no drug in a black box according to the randomized controlled table. The research nurse will be the only person who is not blinded; the research team that collects and analyzes the data, the neurosurgeon who assesses brain relaxation, and the anesthesiologist who administers the anesthesia will be blinded.

### Selection and withdrawal of participants

#### Recruitment

Participants are identified by their presence on surgical lists and are recruited from the tumor wards. The investigator informs the participant or the participant’s nominated representative of all aspects pertaining to participation in the study.

The study intervention will be completed immediately after the surgery, but follow-up visits will extend for three months following surgery. The medical notes will be reviewed following hospital discharge for in-hospital complications and medication use.

#### Inclusion criteria

Patients scheduled to receive elective supratentorial tumor resection at Beijing Tiantan Hospital, Capital Medical University who are older than 18 and younger than 60 years will be included if their preoperative imaging (computed tomography or magnetic resonance imaging) shows a midline brain shift.

#### Exclusion criteria

Patients will be excluded if their American Society of Anesthesiologists physical status score is III or higher or if their Glasgow Coma Scale score is <13. Patients will be excluded if their plasma sodium is <130 mEq/L or >150 mEq/L. Patients will be excluded if they have renal insufficiency or if their creatinine clearance is <30 mL/kg. Patients will also be excluded if they have heart disease or if their cardiac ejection fraction is <20%.

### Informed consent

Written informed consent will be obtained one day before surgery. The capacity for providing informed consent will be assessed routinely by the neurosurgery team, who will decide whether the patient is suitable for inclusion in the study. A member of the research team will also perform an additional assessment of the participant’s ability to provide consent one day prior to starting the study. If either the neurosurgery or research team determines that the patient is unable to give informed consent, the patient’s entrusted agent will be asked to give written informed consent. If either the patient or his entrusted agent declines consent, then the patient will not be entered into the study. Only patients who give informed written consent will be included in the trial. All members of the research team are trained in obtaining informed consent in accordance with good clinical practice.

### Ethics committee and regulatory approval

The trial will be conducted in accordance with the ethical principles outlined in the Declaration of Helsinki, 1996 [14]; National Ethics Review for Biomedical Research Involving Humans (Trial), 2007 [15]; and International Ethical Guidelines for Biomedical Research Involving Human Subjects, 2002 [16]. Approval was obtained from the China Ethics Committee of Registering Clinical Trials on 2 December 2013 (reference number ChiECRCT2013033).

### Study intervention

#### Medical product administration

Patients allocated to the investigational medical product group will receive 20% mannitol at doses of 0.7, 1.0, or 1.4 g/kg at the onset of surgical incision. The drug will be administered intravenously at a rate of 600 mL/h.

The dose and timing of administration of intravenous mannitol will be similar to those described by Quentin et al. [11].

#### Concomitant treatments

Peritumoral brain edema will be evaluated via a magnetic resonance imaging (MRI) scan read by an experienced radiologist and graded using the Steinhoff classification: 0-no signs of edema, I-peritumoral brain edema limited to 2 cm, II-peritumoral brain edema limited to half of the hemisphere, or III-peritumoral brain edema in more than half of the hemisphere [17].

Peripheral venous access will be established upon the patient’s arrival in the operating room. Routine monitoring and data collection will include noninvasive blood pressure, electrocardiograph, pulse oxygen saturation, end-tidal carbon dioxide pressure, exhaled anesthetic concentration, bispectral index, temperature, and urine output. All patients will be premedicated with midazolam (0.05 mg/kg) intravenously 15 min prior to anesthesia induction. Anesthesia will be induced with sufentanil (0.2 to 0.3 μg/kg), propofol (target-controlled infusion with an initial plasma concentration of 4 μg/mL), and rocuronium (0.6 mg/kg). After tracheal intubation, mechanical ventilation will be performed with the following parameters: tidal volume, 8 to 10 mL/kg; respiratory rate, 12 to 15/min; I: E, 1:2; inhaled oxygen fraction, 60%; and flow rate of fresh gas, 1 to 2 L/min to maintain normocapnia. Anesthesia will be maintained with propofol (target-controlled infusion with a plasma concentration of 3 to 5 μg/mL) and remifentanil (0.1 to 0.2 μg/kg/min) supplemented with rocuronium for muscle relaxation. Sufentanil will be injected to attenuate the potent stress responses induced by noxious stimuli at certain time points, such as scalp incision and skull drilling. Crystalloid infusion and/or colloid will be infused as needed to treat intravascular volume depletion. Fluid input and output will be monitored closely.

#### Standard care

Standard care will be identical in both groups; only the administration of mannitol will differ.

#### Cost analysis

Data on the cost of treatment will include standardized costs for physiotherapy, neurosurgery, anesthesia, and postoperative care. Data will be presented in terms of total non-operative costs, costs per day, and excess costs attributable to the treatment group.

#### Laboratory analyses

Blood will be sampled from a peripheral artery prior to the initial administration and 30, 60, 120, and 180 min after the infusion of mannitol to measure the plasma concentration of electrolytes, osmolality, lactic acid, and glucose.

### Statistics

The SPSS 19.0 software package for Windows (SPSS, Inc., Chicago, IL) will be used for all statistical analyses. The primary outcome measure (difference in brain relaxation between groups) in the full analysis will be analyzed using the chi-squared test or Fisher’s exact test. The comparison of continuous variables between groups will be accomplished using a one-way analysis of variance. Any perioperative factors that differ between groups will be tested together with the treatment factor (the administration of mannitol or placebo) via multivariate logistic regression analysis against the brain relaxation of the patients to assess their effects on brain relaxation. A significance level of *P* <0.05 will be chosen for all tests.

#### Sample size and justification

**Figure 1:**
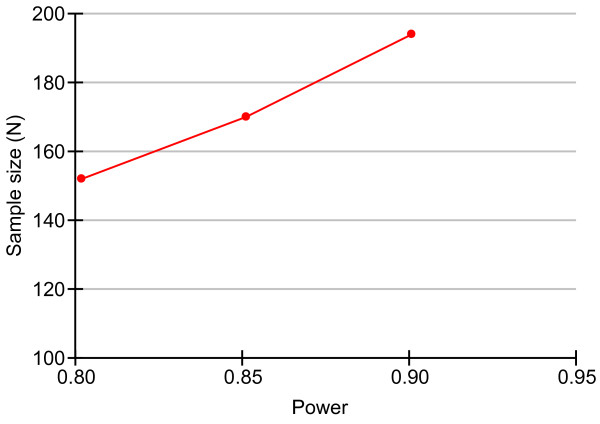
**Sample size calculation.** Power is the probability of rejecting a false null hypothesis; this item should be close to one. N is the size of the sample drawn from the population.

We used the PASS 2008 software (NCSS LLC, USA) for Windows to calculate the sample size. According to the data of incidence of satisfactory brain relaxation in Quentin’s research [11], a sample size of 194 achieves 85% power to detect a significance level (alpha) of 0.05 (Figure 1) using a chi-squared test with nine degrees of freedom [18]. Degree of freedom is the chi-squared distribution. Alpha is the probability of rejecting a true null hypothesis. We propose to study 220 patients, with 55 participants in each group. Twenty-six additional patients are included to account for dropouts during the follow-up visits within three months after the brain tumor surgery.

### Reporting of adverse events

All adverse events will be recorded and closely monitored until resolution or stabilization or until it has been shown that the study treatment is not the cause of the event. Participants will be asked to contact the study site immediately in the case of any serious adverse events. The chief investigator will be informed immediately of any serious adverse events and will determine (in cooperation with the treating medical practitioners) the seriousness and causality of these events.

All treatment-related serious adverse events will be recorded and reported to the research ethics committee as part of the annual reports. Unexpected serious adverse events will be reported to the research ethics committee within the relevant time frames. The chief investigator will be responsible for all adverse event reporting.

### Potential conflicts of interest

The manufacturer of mannitol has no involvement in the design or funding of this study. All drugs are being prescribed from the central pharmacy in Beijing Tiantan Hospital at normal prices. There will be no potential conflicts of interest.

## Discussion

Mannitol is often recommended as the first-choice hyperosmotic drug to treat increased intracranial pressure (ICP) and alleviate brain bulk during intracranial surgery [19]. However, the optimal administration and dosage of mannitol for brain relaxation remain controversial, especially in patients with preoperative increased ICP. A common routine dose is frequently chosen for a given surgical procedure regardless of the size of the lesion and the mass effect that results.

Previous prospective randomized trials have focused on the relationship between mannitol and intraoperative brain relaxation; however, none of these trials examined how different doses of mannitol influenced brain relaxation in patients with a preoperative brain midline shift. Rozet et al. [9] observed the effect of a single mannitol dose on brain relaxation in patients with widely varying intracerebral pathologies, including supratentorial tumor. Quentin et al. [11] examined the effects of two doses (0.7 and 1.4 g/kg) of mannitol on brain relaxation in tumor patients, neither of which was the routine clinical dose (1 g/kg); additionally, they did not consider the effect of preoperative intracranial mass. However, these studies indicated that the effect of mannitol on brain relaxation was dose-dependent. The current proposal is a prospective randomized controlled study that aims to examine the effects of three doses of mannitol on brain relaxation in patients with preoperative midline shift.

Patients with large brain lesions have often received repeated mannitol doses to prevent brain herniation; however, the repeated intraoperative use of large doses of mannitol may precipitate serious side effects. The main side effects of mannitol include electrolyte abnormalities (for example, hypokalemia) and renal and cardiac dysfunction. Patients with heart failure, pulmonary edema, electrolyte imbalance, chronic hypertension, coronary heart disease, and diabetes often have renal dysfunction without clinical manifestations. Mannitol can exacerbate this dysfunction and lead to kidney injury [20,21]. Cardiac preload and central venous pressure increase 5 to 15 min after mannitol is administered [22]. The diuretic effects of mannitol may cause water and electrolyte imbalances, hypotension, and decreased plasma concentrations of sodium, potassium, and chlorine [23]. As shown in other studies [24,25], increased doses of mannitol resulted in an increase in osmolarity, a decrease in serum sodium concentration, and an increase in urine output. The development of hyponatremia can be explained by the changes in osmolarity and the initial volume shift toward the intravascular compartment along the osmolar gradient and the resulting hemodilution. Higher doses of mannitol result in a dose-related increase in osmolarity, as well as a similar dose-related decrease in brain water content; this results in better relaxation scores in patients with traumatic brain injury [26]. Manninen et al. [23] described a significant increase in the serum potassium level, which reached a maximum mean increase of 1.5 mmol/L, after high-dose mannitol (2 g/kg) administration in seven patients undergoing cerebral aneurysm clipping. Therefore, patients should be closely and carefully observed for side effects, especially patients who receive a large dose of mannitol and/or have preoperative increased intracranial pressure.

During the operation, the neurosurgeon will decide whether to initiate treatment for brain bulk via the assessment of intraoperative brain relaxation, which is not an objective sign, such as intracranial pressure. Treatments for improving brain relaxation include adjusting the ventilator to induce hyperventilation, expanding the surgical incision site, and infusing mannitol; these procedures contribute to tumor exposure and excision. Thus, the subjective assessment of the degree of brain relaxation can be used as a diagnostic criterion prior to treatment. Monitoring intracranial pressure or cerebral water content is not routine during brain tumor resection because the extra invasive monitoring of patients increases the risk of brain herniation. Sorani et al. [8] performed a retrospective study to characterize the dose–response relationship between mannitol and ICP in intensive care unit patients with traumatic brain injury and found that the degree and incidence of peritumoral edema greatly varied. Therefore, it is not appropriate to measure the effect of mannitol in patients with supratentorial tumors, including gliomas and meningiomas, using brain water content.

In this prospective randomized trial, we will observe the effects of different doses of mannitol on patient outcomes three months postoperatively. Patients undergoing craniotomy commonly have complications that include postoperative cerebral edema, cerebral hemorrhage, recurrence, and even death, which are closely related to intraoperative tumor exposure, resection, and sufficient coagulation. The incidence of postoperative complications determines the length of intensive care unit and hospital stay, as well as patient outcome. Mannitol may help improve tumor exposure and resection. However, if the BBB is damaged, mannitol will be transferred from the ruptured or highly permeable blood vessels into brain tissue, which reverses the osmotic pressure difference and results in brain edema. Animal studies [26] demonstrate that five repeated doses of mannitol lowered ICP and reduced cerebral edema; however, edema increased following greater exposure to mannitol.

## Trial status

The study was also registered with the registry website http://www.chictr.org with the registration number ChiCTRTRC13003984 (17 December 2013). The study began on 2 January 2014, and the planned completion date is 1 April 2015.

## Abbreviations

BBB: blood–brain barrier; ICP: intracranial pressure.

## Competing interests

The authors did not and do not receive any reimbursement or financial benefits and declare that they have no competing interests.

## Authors’ contributions

YP was involved in conception and design, data collection and analysis, manuscript writing, and final approval of the manuscript. XL was involved in conception and design, data collection, manuscript revision, and final approval of the manuscript. AW was involved in conception and design, data collection, manuscript revision, and final approval of the manuscript. RH was involved in conception and design, data analysis, manuscript revision, and final approval of the manuscript. All authors have read and approved the final manuscript.

## Authors’ information

Yuming Peng and Xiaoyuan Liu are co-first authors.
